# Polypyrrole-Derived Nitrogen-Doped Tubular Carbon Materials as a Promising Cathode for Aqueous Aluminum-Ion Batteries

**DOI:** 10.3390/polym16233276

**Published:** 2024-11-25

**Authors:** Xiaoming Zhou, Xiaolei Li, Jiaming Duan, Lihao Zhang, Xinyu Mo, Qing Wu, Yang Liu, Guohui Yuan, Miaosen Yang

**Affiliations:** 1 School of Chemical Engineering, Northeast Electric Power University, Jilin 132012, China; zhouxiaoming0123@163.com (X.Z.); 15169143712@163.com (X.L.); 16602807218@163.com (J.D.); 17356573994@163.com (L.Z.); 15243208066@163.com (X.M.); w15552349281@163.com (Q.W.); 2 School of Chemistry and Chemical Engineering, Harbin Institute of Technology, Harbin 150001, China

**Keywords:** aluminum-ion battery, cathode material, nitrogen-doped tubular carbon, aqueous, electrolyte

## Abstract

The advantages of aluminum-ion batteries in the area of power source systems are: inexpensive manufacture, high capacity, and absolute security. However, due to the limitations of cathode materials, the capacity and durability of aluminum-ion batteries ought to be further advanced. Herein, we synthesized a nitrogen-doped tubular carbon material as a potential cathode to achieve advanced aqueous aluminum-ion batteries. Nitrogen-doped tubular carbon materials own an abundant space (367.6 m^2^ g^−1^) for electrochemical behavior, with an aperture primarily concentrated around 2.34 nm. They also exhibit a remarkable service lifespan, retaining a specific capacity of 78.4 mAh g^−1^ at 50 mA g^−1^ after 300 cycles. Additionally, from 2 to 300 cycles, the material achieves an appreciable reversibility (coulombic efficiency CE: 99.7%) demonstrating its excellent reversibility. The tubular structural material possesses a distinctive hollow architecture that mitigates volumetric expansion during charging and discharging, thereby preventing structural failure. This material offers several advantages, including a straightforward synthesis method, high yield, and ease of mass production, making it highly significant for the research and development of future aluminum-ion batteries.

## 1. Introduction

Lithium-ion storage devices are prevalent in everyday applications that profit from their high capacity, compact size, and portability. However, they also present significant drawbacks, including their high cost and safety concerns [1]. In contrast, aluminum-ion batteries offer advantages such as lower cost, abundant material availability, and enhanced safety, positioning them as a viable option for making progress in progressive battery technologies [2,3,4]. Unfortunately, despite these advantages, cathode materials, with their poor electrochemical performance, seriously hinder the development of aluminum-ion storage devices [5,6,7]. Consequently, enhancing the capabilities of aluminum-ion storage devices should preferentially promote the progress of cathode materials [8,9,10].

Activated carbon, carbon nanotubes, graphene, fullerene, and other carbon materials characterized by high stability and conductivity represent one of the most promising cathode materials in emerging aluminum-ion storage systems [11,12,13,14]. Among them, graphene-like materials demonstrate exceptional performance associated with superior specific capacity and high charge and discharge plateau potentials, as well as a durable service lifespan [15,16,17]. In 2015, Hongjie Dai and co-authors first proposed a stable aluminum-ion battery utilizing pyrolytic graphite as the cathode together with ionic liquid AlCl_3_/[EMIM]Cl electrolyte, thereby establishing a spic-and-span research avenue in the area of aluminum-ion storage systems [18]. The synthesized high-porosity three-dimensional graphite foam exhibits a reserved specific capacity of 60 mAh g^−1^ over 7500 cycles at 4 A g^−1^. Researchers primarily enhance Al^3+^ ion storage behavior and transmission kinetics via modifying diversiform intrinsic structures and characteristics of carbon-based materials [19]. Prof. Bao’s research team put forward that C_70_ fullerene materials were a possible active species for use in aluminum-ion storage systems since they exhibit a skyscraping reversible specific capacity of 750 mAh g^−1^ at 200 mA g^−1^ as well as a high working potential of approximately 1.65 V [20]. The exceptionally stable zero-dimensional structure of fullerene particles prevents degradation during charge and discharge cycles, resulting in excellent cycle stability. To further enhance the ion storage capacity of aluminum-ion storage materials, the strategies that are usually employed involve building up more contact and transport space between active materials and electrolyte ions, integrating with heterogeneous species, as well as increasing the lattice spacing and improving the lattice stability. For instance, Zhihui Dai’s research team designed good-looking carbon octahedrons equipped with nitrogen-atom doping and porous architecture, which were prepared using a metal organic framework precursor [21]. The carbon octahedron’s cathode within the ionic liquid electrolyte showed impressive aluminum-ion storage performance owing to the positive cooperativity of its structural nature and componential peculiarity. For developing performance-enhanced aluminum-ion storage materials, the significant ways for fabricating the carbon-based cathode materials are to engineer abundant ion transfer space and active sites [22]. This is pivotal for achieving cost-effective aluminum-ion battery materials and maintaining a settled dynamic host, as well as ensuring preeminent ion storage behaviors.

Furthermore, the presence of hydroxyl/epoxy and carbonyl/carboxyl ingredients at the borders of graphene flakes facilitates the storage behaviors for numerous ion clusters, thereby positively affecting the electrochemical properties of aluminum-ion kinetics [23]. Zhang et al. utilized 4-aminophenol as both a reducing agent and nitrogen-dopant to synthesize partially reduced graphene oxide materials with a high hydroxyl content, achieving a remarkable 6.15% [24]. The resulting sample at 0.3 A g^−1^ exhibited a brilliant storage ability of 284.1 F g^−1^, maintaining 228.4 F g^−1^ despite at 10 A g^−1^. Recent research has actively focused on aluminum-ion storage systems utilizing ionic liquids as charge transport media [25,26,27]. Ionic liquids exhibit an excellent thermodynamic steady quality and a wide operating potential, as well as a high ion transport property. In recent years, electrolytes comprising AlCl_3_/[EMIM]Cl have gained widespread application in aluminum-ion storage systems [28,29]. However, the high cost, flammability, and low energy storage efficiency of ionic liquid electrolytes hinder their practical applications [30,31]. In contrast, aqueous aluminum-ion storage systems offer advantages, for instance, inexpensive manufacture, environmental sustainability, and non-flammability, making them suitable candidates for commercial power source markets [32,33].

In this work, nitrogen-doped carbon tubular materials were synthesized by a green sacrificial template method together with carbonization treatment. The methyl orange was used as a tubular template for the polypyrrole precursor, and it can be facilely removed within hot water in a subsequent process. The nitrogen-doped carbon tubular materials were obtained after the calcination treatment of the tubular polypyrrole precursor. Benefitting from the structural advantage, these nitrogen-doped tubular carbon cathode materials used in aluminum-ion storage systems deliver impressive rate and cycling properties within an aqueous AlCl_3_ electrolyte. Specifically, the maximum specific capacity of a tubular carbon cathode achieves 78.4 mAh g^−1^ after 300 cycles at 50 mA g^−1^, maintaining 24.5 mAh g^−1^ at 800 mA g^−1^. The morphological and electrochemical analysis results indicate that the carbon tubular materials exhibit not only high quality and efficiency but also excellent electrochemical properties, making them a prospective material applying to aluminum-ion battery cathodes.

## 2. Materials and Methods

### 2.1. Synthesis of Nitrogen-Doped Tubular Carbon Materials

The experiment utilized three raw materials: methyl orange, ferric chloride, and pyrrole, all sourced from Yongda Reagent, Tianjin, China. Initially, 0.4 g of methyl orange (≥96%) was placed into 120 mL of ultrapure water. The addition of 420 µL of pyrrole monomer (99%) was subsequently placed into the above dispersion. This mixture was agitated for 30 min to ensure uniformity. Subsequently, 0.972 g of anhydrous ferric chloride (99%) was incorporated into the dispersion, which was then agitated for 24 h, resulting in a black suspension [34,35]. The black precursors were collected through vacuum filtration and rinsed multiple times with hot ultrapure water to wipe off any excess methyl orange. The black polypyrrole powder was desiccated in an oven at 80 °C for 12 h. Finally, the polypyrrole precursor underwent a sintering process in an argon atmosphere for approximately 5 h, with a heating rate of 5 °C per minute up to 900 °C, to result in nitrogen-doped tubular carbon materials.

### 2.2. Material Characterizations

The valence states of carbon, oxygen, and nitrogen ingredients in the tubular carbon materials were analyzed with an X-ray photoelectron spectrometer (XPS, ESCALAB 250Xi, Thermo Scientific™, Madison, WI, USA). The tubular feature was examined using scanning electron microscopy (SEM) with a ZEISS Merlin Compact instrument (GeminiSEM 300, Oberkochen, Germany). Element distribution within the tubular carbon materials was analyzed through energy dispersive spectroscopy (EDS). The microscopic and internal structures of the tubular carbon materials were assessed using transmission electron microscopy (TEM, JEM-2100, JEOL, Tokyo, Japan). To determine the specific surface area and aperture distribution, nitrogen adsorption-desorption tests were conducted using a Micromeritics ASAP2460, Norcross, GA, USA. Additionally, X-ray diffraction (XRD, Bruker D2, Billerica, MA, USA) was utilized to investigate the crystal structure of the tubular carbon materials.

### 2.3. Electrochemical Measurements

The ion storage behavior of the tubular carbon materials at ambient temperature was evaluated using a CR2032 button cell. This cell employed an aluminum slice as the anode, a glass fiber membrane as the diaphragm, and a 1 M aluminum trichloride aqueous solution as the electrolyte. An even, granular slurry was obtained by mixing the tubular carbon material, acetylene black, and polyvinylidene fluoride (PVDF) in a percentage of 70:20:10, and it was subsequently applied to carbon paper. The nitrogen-doped tubular carbon material electrode was formed by drying this mixture in a blast oven at 120 °C for 12 h. The electrolyte was prepared at room temperature in an open environment. Specifically, 4.8 g of aluminum chloride powder (Aladdin Reagent (Shanghai) Co., LTD., Shanghai, China, AR, 97%) was placed in 20 mL of ultrapure water to achieve the desired 1 mol L^−1^ aluminum chloride solution, which was stirred until it became transparent. The cyclic voltammetry (CV) test was conducted using a CHI650E electrochemical analyzer. The specific capacity, cycle stability, and rate performance of the battery were assessed on the Neware galvanostatic testing device (CT4008A, NEWARE TECHNOLOGY LIMITED, Shenzhen, China). The potential scope for galvanostatic charge–discharge (GCD) and CV tests was established between 0.2 and 1.6 V.

## 3. Results and Discussion

**Figure 1:**
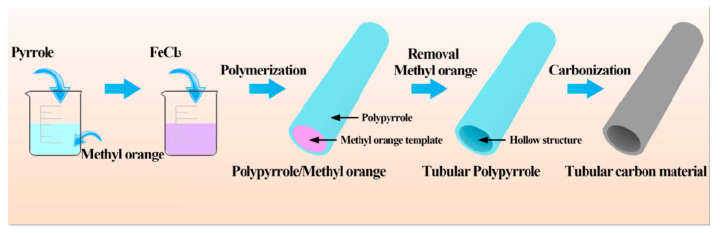
Schematic diagram of the fabrication process of tubular carbon materials.

The fabrication procedure illustration for nitrogen-doped porous tubular carbon material is depicted in Figure 1 Initially, during the dispersion of methyl orange in the water, methyl orange molecules would self-assemble to form one-dimensional microrods micelles according to the reported literature [36,37]. The methyl orange serves as an initiator and a hard template for the polymerization of pyrrole monomer. Subsequently, the pyrrole monomer polymerized on the surface of one-dimensional methyl orange microrods. After removing the methyl orange, polypyrrole nanotubes can be obtained. Finally, the nitrogen-doped tubular carbon materials were prepared after the carbonating process of polypyrrole nanotubes.

**Figure 2:**
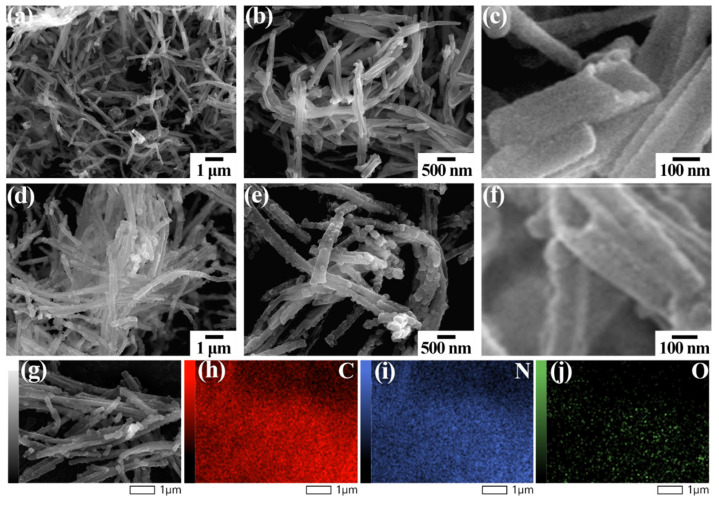
(**a**–**c**) SEM image for tubular polypyrrole polymer precursor. (**d**–**f**) SEM image of tubular carbon material. (**g**–**j**) EDS mapping images of tubular carbon material.

The different regional SEM images of the tubular polypyrrole polymer precursor obtained after removing the methyl orange template are shown in Figure 2a and Figure S1a. The images reveal a substantial quantity of tubular materials throughout the field of view, indicating the high efficiency of this unique approach. Figure 2b illustrates that the precursor exhibits a smooth surface with dimensions of approximately 200 nm. The length range of the tubular polypyrrole polymer precursor is from approximately 1.0 μm to 5.0 μm. Furthermore, Figure 2c demonstrates a distinct internal opening structure, confirming the presence of an internal hollow configuration. The different regional SEM images of the carbonized tubular polypyrrole polymer precursor are depicted in Figure 2d and Figure S1b. The produced carbon materials reveal a uniform tubular structure, suggesting that the carbonization process did not destroy the hollow tubular structure of the polypyrrole polymer precursor. As shown in Figure 2e, the sample inherited the tubular architecture of the polymer precursor. The tubular carbon material displays a uniform morphology, measuring approximately 200 nm in diameter. Additionally, the interior of the material maintains a hollow structure, as shown in Figure 2f. This tubular configuration is well suited for efficiently accommodating aluminum ions and facilitating ion transport. The element mapping images in Figure 2g–j indicate that C, N, and O ingredients are evenly distributed throughout the tubular carbon material. Notably, the nitrogen content reaches up to 4.45 atom%, and the elemental contents of carbon and oxygen are 88.23 atom% and 7.33 atom%, as illustrated in Figure S2.

**Figure 3:**
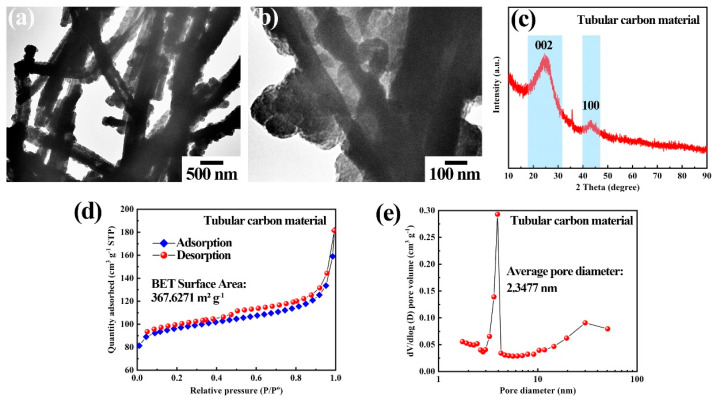
(**a**,**b**) The different TEM images of hollow tubular carbon material. (**c**) XRD pattern of tubular carbon material. (**d**) Nitrogen adsorption-desorption isotherms, and (**e**) aperture distribution of tubular carbon material.

The TEM images presented in Figure 3a,b further corroborate that the material possesses a hollow structure, which significantly mitigates the volume expansion associated with the insertion and removal of ions during frequent dynamic electrochemical behaviors. The crystal structure of the tubular carbon material was evaluated using X-ray diffraction, as illustrated in Figure 3c. The loose signal located at 25.6° corresponds to the (002) crystal plane diffraction, indicating the disorder within the carbon material [38]. Additionally, the BET surface area and aperture structure of the tubular carbon materials were evaluated using nitrogen adsorption and desorption tests. The resulting adsorption and desorption curves in Figure 3d are inconsistent and exhibit typical type IV isotherms, implying the emergence of mesopores within tubular carbon material [39]. Basing on classical BET model calculations, the specific surface area of the material can reach 367.6 m^2^ g^−1^. The aperture range analysis, depicted in Figure 3e, reveals a concentration of pore sizes around 2.34 nm. The abundance of mesopores in the material facilitates electrolyte penetration and ion transfer, while the extensive surface area offers additional adsorption sites for electrolyte ions.

**Figure 4:**
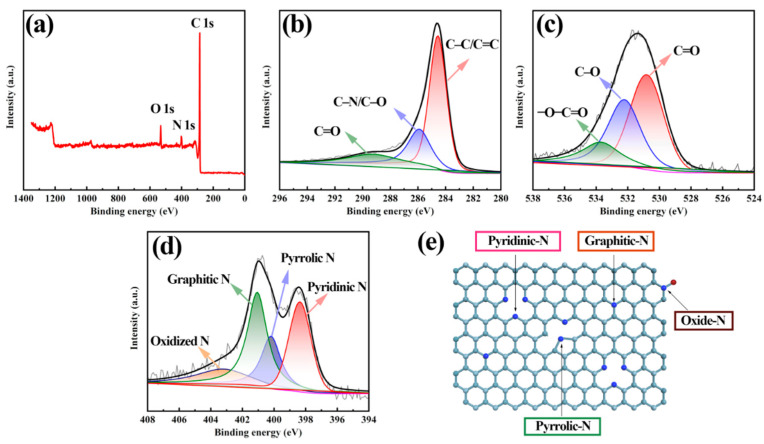
(**a**) XPS element full spectrum of tubular carbon material. Regional XPS spectra of (**b**) C 1s, (**c**) O 1s and (**d**) N 1s. (**e**) Structural illustration for nitrogen-doped carbon materials.

The chemical ingredient and valence state of tubular carbon material were detected using XPS technology. The XPS full spectrum, as shown in Figure 4a, indicates that tubular carbon material predominantly consists of C, O and N ingredients. The presence of nitrogen may be attributed to residual nitrogen from the calcination of polypyrrole, while the oxygen signal likely originates from trace amounts of air incorporated during the calcination process. The XPS results for the N 1s and O 1s spectra were relatively intense, suggesting effective incorporation of nitrogen and oxygen into the carbon material. Importantly, no impurity elements beyond carbon, oxygen, and nitrogen were detected. Figure 4b illustrates that the C 1s spectrum can be assign to three distinct groups, with signals observed at 284.8 eV, 286.0 eV, and 289.8 eV, corresponding to C-C/C=C, C-O/C-N, and C=O, respectively [40]. The detailed XPS spectrum of O 1s, presented in Figure 4c, reveals three distinct components: C=O at 531.2 eV, C-O at 532.4 eV, and O-C=O at 533.6 eV. Oxygen doping primarily occurs at the surface and defects of the tubular carbon materials, effectively modulating the electronic structure and enhancing electrical conductivity [21]. The incorporation of oxygen also increases the surface energy of the tubular carbon material, thereby improving its affinity for the electrolyte. Similarly, the N1s spectra depicted in Figure 4d might be dissociated into four distinct signals centered at 398.4 eV, 400.1 eV, 401.2 eV, and 403.2 eV, pertaining to pyridine-N, pyrrole-N, graphite-N, and oxide-N, respectively [41]. The sp^2^ hybridized pyridine-N and graphite-N atoms enhance electronical conductivity for the carbon matrix by modulating the electron donor and acceptor properties [42], as illustrated in Figure 4e. This modulation subsequently improves electrochemical performance by facilitating both electron transport and ion diffusion. Pyridine-N, which substitutes a carbon atom at the border or defect of the primary carbon lattice, acts as an electrochemically active site characterized by a low energy barrier. The nitrogen doping content in this tubular carbon material, determined through XPS analysis, was found to be 6.25 atom%, as shown in Figure S3. This nitrogen doping significantly contributes to enhanced electrical conductivity and reactivity. Meanwhile, the carbon content and oxygen content are 87.7 atom% and 6.05 atom%, respectively. The results of the XPS elemental analysis basically agree with the results of the SEM-EDS analysis for nitrogen-doped tubular carbon materials.

**Figure 5:**
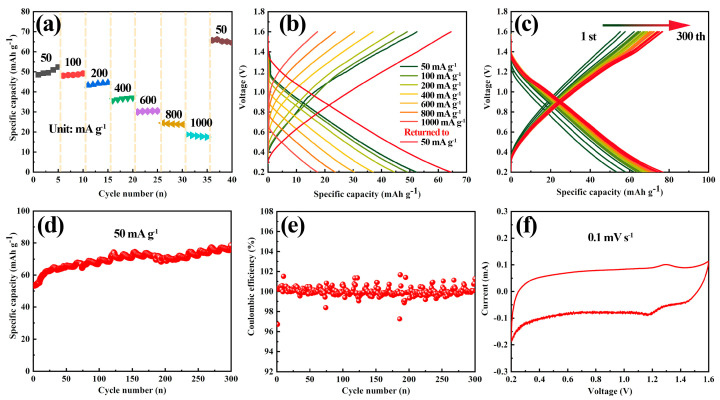
(**a**) Rate properties and (**b**) GCD curves of tubular carbon material at various operating rates. (**c**) GCD curves during cycling at 50 mA g^−1^. (**d**) Cycling lifespan and (**e**) coulombic efficiency of tubular carbon material at an operating rate of 50 mA g^−1^. (**f**) CV curve of tubular carbon material recorded at a test rate of 0.1 mV s^−1^.

The rate abilities of the nitrogen-doped tubular carbon material across operating rates within scope from 50 to 1000 mA g^−1^ are illustrated in Figure 5a. At these operating rates—specifically 50, 100, 200, 400, 600, 800, and 1000 mA g^−1^—the discharge capacity of tubular carbon material consistently reaches 52.3 mAh g^−1^, 49.2 mAh g^−1^, 44.6 mAh g^−1^, 37.1 mAh g^−1^, 30.5 mAh g^−1^, 24.5 mAh g^−1^ and 18.8 mAh g^−1^. Remarkably, when the operating rate is reverted to 50 mA g^−1^, the discharge capacity is restored to 66.1 mAh g^−1^, indicating a notable enhancement in contrast to the initial capacity. The results declare that the structure of the tubular carbon material remains stable and does not collapse, even after undergoing high-current density charge and discharge cycles [43]. Following the high-current charge–discharge test, the material exhibits a stable charge–discharge curve characterized by minimal polarization, as illustrated in the red GCD curves in Figure 5b. Subsequently, a cycle life analysis was carried out at an operating rate of 50 mA g^−1^, as presented in Figure 5c. Over 300 cycles, the profile of GCD curves showed minimal changes, indicating good reversibility [44]. The ion storage capacity of tubular carbon material demonstrates a gradual increasing trend, as exhibited in Figure 5d. The initial discharge capacity of the tubular carbon material was 52.3 mAh g^−1^, and after 300 cycles it increased to 78.4 mAh g^−1^. In comparison, untreated commercial carbon nanotubes were employed in comparative experiments, as presented in Figure S4. The results reveal that the initial discharge capacity of carbon nanotubes was only 6.8 mAh g^−1^. After 2 cycles, the capacity remained below 5 mAh g^−1^, suggesting that the carbon nanotubes are unable to reversibly insert and extract cations.

In addition, Table 1 shows the comparison of the electrochemical performance of partial porous or N-doped carbon-based cathode materials for aluminum-ion batteries. It can be seen that compared with that of reported porous or N-doped carbon-based cathode materials for aluminum-ion batteries, the cycling stability and specific capacity of our tubular carbon materials are located at a higher level despite using the low-cost aqueous electrolyte. Meanwhile, it is worth noting that in contrast to a non-aqueous ionic liquid electrolyte, such as the widely used AlCl_3_/EMImCl, the AlCl_3_ aqueous electrolyte is low-cost and does not need expensive glove boxes to assemble the batteries. Therefore, aqueous electrolyte is in more favor for practical and commercial aluminum-ion batteries, implying the useful significance of developed polypyrrole-derived nitrogen-doped tubular carbon materials.

The coulombic efficiency (CE) of the tubular carbon material is presented in Figure 5e. The initial coulombic efficiency recorded was 96.7%, while the efficiency of the second cycle increased significantly to ~100%. Over cycles 2 to 300, the average CE of the material reached 99.7%, indicating that the material demonstrates good reversibility and repeatability [45]. Figure 5f presents the CV curve of the aluminum-carbon storage system, collected at a test rate of 0.1 mV s^−1^ within an operating scope of 0.2 to 1.6 V. Within this voltage range, the CV curve does not exhibit distinct oxidation and reduction peaks; rather, it displays a quasi-rectangular shape, characteristic of typical capacitive response behavior. This observation suggests that the carbon material primarily contributes to capacity through its capacitive properties. Furthermore, the symmetrical trend observed in the CV curve indicates that the battery undergoes reversible ion insertion and extraction reactions. Because aluminum chloride is an acidic salt solution and the pH value of 1.0 mol L^−1^ AlCl_3_ of aqueous solution is about 1.3, the cations in electrolyte contain Al^3+^ and H^+^ [46]. Therefore, according to the reported literature, Al^3+^ and H^+^ may undergo co-insertion and de-insertion on tubular carbon materials during charge and discharge processes [47].

**Figure 6:**
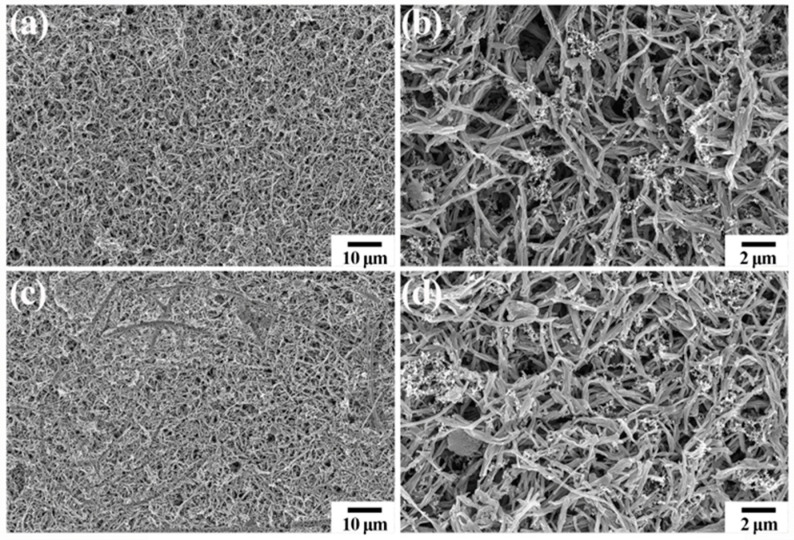
(**a**,**b**) SEM images of fresh nitrogen-doped tubular carbon electrode. (**c**,**d**) SEM images of nitrogen-doped tubular carbon electrode after rate capability test.

In order to demonstrate the structural stability of nitrogen-doped tubular carbon electrodes, the surface morphology of the nitrogen-doped tubular carbon cathodes at a fresh state and after a rate capability test are shown in Figure 6. Figure 6a exhibits the low magnification SEM image of a fresh electrode, where the overall surface morphology of the electrode is uniform with a porous structure, and no aggregates can be found. Furthermore, the high magnification SEM image of a fresh electrode in Figure 6b clearly displays the even dispersion of nitrogen-doped tubular carbon materials. The tubular structure of active carbon materials is hardly destroyed during electrode preparation. Figure 6c,d shows the SEM images of the electrode after a rate capability test and it presents denser surface morphology in contrast to the fresh electrode, which may be due to the volumetric change of tubular carbon materials during cycling. Notably, the morphology and structure of tubular carbon materials show no significant damage compared to that of materials in the fresh electrode, indicating the stable construction during the charging and discharging processes.

**Table 1:** Comparison of the electrochemical performance of carbon-based cathode materials for aluminum-ion batteries.

Sample	Electrolyte	Maximal Specific Capacity	Specific Capacity After Cycling	Ref.
Hierarchical porous carbon octahedrons	AlCl_3_/urea	~60.8 mAh g^−1^ (0.1 A g^−1^)	60.8 mAh g^−1^ (0.1 A g^−1^, 200 cycles)	[21]
Nitrogen doped three-dimensional porous carbon material	AlCl_3_/acetamide	33 mAh g^−1^ (0.5 A g^−1^)	23 mAh g^−1^ (0.5 C, 20 cycles)	[48]
Porous activated carbon derived from coconut shell chars	AlCl_3_/EMImCl	150 mAh g^−1^ (0.1 A g^−1^)	80 mAh g^−1^ (1.0 A g^−1^, 1500 cycles)	[49]
Synthetic graphite flakes	AlCl_3_/EMImCl	85 mAh g^−1^ (1 C)	100 mAh g^−1^ (15 C, 1600 cycles)	[50]
MOF-derived carbon	AlCl_3_/EMImCl	282.1 mAh g^−1^ (0.5 A g^−1^)	110 mAh g^−1^ (1.0 A g^−1^, 1000 cycles)	[32]
Polydopamine-derived N-doped carbon nanospher	AlCl_3_/EMImCl	118 mAh g^−1^ (1.0 A g^−1^)	65 mAh g^−1^ (1.0 A g^−1^, 5500 cycles)	[51]
Amorphous carbon materials	AlCl_3_/EMImCl	63.6 mAh g^−1^ (0.2 A g^−1^)	55.5 mAh g^−1^ (0.2 A g^−1^, 1000 cycles)	[52]
Twisted CNT yarn	AlCl_3_/EMImCl	102 mAh g^−1^ (0.4 A g^−1^)	53.9 mAh g^−1^ (2.0 A g^−1^, 260 cycles)	[53]
Polypyrrole-derived nitrogen-doped tubular carbon materials	AlCl_3_/aqueous	78.4 mAh g^−1^ (0.05 A g^−1^)	78.4 mAh g^−1^ (0.05 A g^−1^, 300 cycles)	This work

Polypyrrole-derived nitrogen-doped tubular carbon materials demonstrate remarkable performance, which is primarily due to the following key advantages. (1) The hollow structure of these tubular carbon materials offers an ample reaction surface area for the accommodation and transfer of cations, facilitating swift ion transfer kinetics. (2) The incorporation of nitrogen species leads to the creation of numerous defects in carbon substances derived from polypyrrole, which increases the availability of cation storage sites and boosts the electronic conductivity of tubular carbon materials, thereby enhancing the specific capacity. (3) The hollow tubular carbon materials exhibit superior dispersion properties and anti-agglomeration ability. Consequently, the electrode fabricated by this tubular carbon material also possesses excellent dispersion and porosity, which could promote effective electrolyte wetting and ion transport, contributing to boost rate performance. In addition, the raw materials required for the preparation of tubular carbon materials are low-cost and nontoxic. The synthetic process is relatively straightforward and highly efficient. Meanwhile, tubular carbon materials exhibit excellent stability in terms of cycling property. All the above merits indicate that the polypyrrole-derived nitrogen-doped tubular carbon materials have potential for large-scale production and application in aluminum-ion storage systems.

## 4. Conclusions

This work effectively demonstrates the large-scale preparation of nitrogen-doped tubular carbon materials utilizing methyl orange as a sacrificial template. Scanning electron microscopy images reveal a high yield of tubular carbon material, while transmission electron microscopy images illustrate a distinct hollow structure within the material. The tubular carbon materials exhibit an ample space (367.6 m^2^ g^−1^) for electrolyte ion transport, with an aperture primarily around 2.34 nm. At a charge and discharge rate of 50 mA g^−1^, a tubular carbon material cathode could achieve steady operation over 300 cycles, maintaining a reversible capacity of 78.4 mAh g^−1^. Throughout the cycling process, GCD curves demonstrate minimal variation, certifying that the tubular carbon materials possess excellent cyclic reversibility. The morphological analysis based on the electrode at a fresh state and after cycling demonstrates the stable structure of tubular carbon materials. These findings underscore the importance of nitrogen doping and microstructure as critical units affecting the ion storage behavior of carbon-based materials. Given the advantages of a simple preparation method, inexpensive manufacture, safety, and nontoxicity, nitrogen-doped tubular carbon materials present great potential for serving as cathodes in aluminum-ion storage systems.

## Data Availability

Data will be made available on request.

## Supplementary Materials

The following supporting information can be downloaded at: https://www.mdpi.com/article/10.3390/polym16233276/s1, Figure S1. (a) SEM image for tubular polypyrrole polymer precursor; (b) SEM image of tubular carbon material. Figure S2. SEM-EDS elemental analysis of nitrogen-doped tubular carbon materials. Figure S3. XPS elemental analysis of nitrogen-doped tubular carbon materials. Figure S4. Electrochemical performance of untreated commercial carbon nanotubes as the cathode for aluminum-ion battery. (a) Typical GCD curve, (b) cycling performance at 50 mA g^−1^.

## References

[1] Khan S.J., Alkhedher M., Raza R., Ahmad M.A., Majid A., Din E. (2022). Electrochemical Investigation of PANI:PPy/AC and PANI:PEDOT/AC Composites as Electrode Materials in Supercapacitors. Polymers.

[2] Meng H., Ran Q., Zhu M.H., Zhao Q.Z., Han G.F., Wang T.H., Wen Z., Lang X.Y., Jiang Q. (2024). Benzoquinone-Lubricated Intercalation in Manganese Oxide for High-Capacity and High-Rate Aqueous Aluminum-Ion Battery. Small.

[3] Lv A., Wang M., Shi H., Lu S., Zhang J., Jiao S. (2023). A Carbon Aerogel Lightweight Al Battery for Fast Storage of Fluctuating Energy. Adv. Mater..

[4] Wang S., Huang S., Yao M., Zhang Y., Niu Z. (2020). Engineering Active Sites of Polyaniline for AlCl_2_^+^ Storage in an Aluminum-Ion Battery. Angew. Chem. Int. Ed..

[5] Zhang H., Liu Y., Yang L., Zhao L., Dong X., Wang H., Li Y., Sun T., Li Q., Li H. (2022). Evidence for dual anions co-insertion in a transition metal chalcogenide cathode material NiSe_2_ for high-performance rechargeable aluminum-ion batteries. Energy Storage Mater..

[6] Pan W., Zhao Y., Mao J., Wang Y., Zhao X., Leong K.W., Luo S., Liu X., Wang H., Xuan J. (2021). High-Energy SWCNT Cathode for Aqueous Al-Ion Battery Boosted by Multi-Ion Intercalation Chemistry. Adv. Energy Mater..

[7] Zhang C., Gu H., Hu Y., Zhang W., Li Z. (2023). Investigation on the energy storage performance of Cu_2_Se@MnSe heterojunction hollow spherical shell for aluminum-ion battery. Chem. Eng. J..

[8] Li R., Xu C., Wu X., Zhang J., Yuan X., Wang F., Yao Q., Balogun M.S., Lu Z., Deng J. (2022). Aluminum-ion storage reversibility in a novel spinel Al_2_/_3_Li_1/3_Mn_2_O_4_ cathode for aqueous rechargeable aluminum batteries. Energy Storage Mater..

[9] Zhuang R., Huang Z., Wang S., Qiao J., Wu J.-C., Yang J. (2021). Binder-free cobalt sulfide@carbon nanofibers composite films as cathode for rechargeable aluminum-ion batteries. Chem. Eng. J..

[10] Jiang J., Li H., Huang J., Li K., Zeng J., Yang Y., Li J., Wang Y., Wang J., Zhao J. (2017). Investigation of the Reversible Intercalation/Deintercalation of Al into the Novel Li_3_VO_4_@C Microsphere Composite Cathode Material for Aluminum-Ion Batteries. ACS Appl. Mater. Interfaces.

[11] Autthawong T., Ratsameetammajak N., Khunpakdee K., Haruta M., Chairuangsri T., Sarakonsri T. (2024). Biomass Waste Utilization as Nanocomposite Anodes through Conductive Polymers Strengthened SiO_2_/C from Streblus asper Leaves for Sustainable Energy Storages. Polymers.

[12] Liu Z., Wang J., Ding H., Chen S., Yu X., Lu B. (2018). Carbon Nanoscrolls for Aluminum Battery. ACS Nano.

[13] Debnath S., Phan C., Searles D.J., Hankel M. (2020). Graphdiyne and Hydrogen-Substituted Graphdiyne as Potential Cathode Materials for High-Capacity Aluminum-Ion Batteries. ACS Appl. Energy Mater..

[14] He Y., Han X., Du Y., Zhang B., Xu P. (2016). Heteroatom-Doped Carbon Nanostructures Derived from Conjugated Polymers for Energy Applications. Polymers.

[15] Xu C., Luo X. (2022). First-Principles Investigation of Graphenylene as a Long-Life Cathode Material in Aluminum Ion Batteries. ACS Appl. Energy Mater..

[16] Huang X., Liu Y., Zhang H., Zhang J., Noonan O., Yu C. (2017). Free-standing monolithic nanoporous graphene foam as a high performance aluminum-ion battery cathode. J. Mater. Chem. A.

[17] Huang H., Zhou F., Shi X., Qin J., Zhang Z., Bao X., Wu Z.-S. (2019). Graphene aerogel derived compact films for ultrafast and high-capacity aluminum ion batteries. Energy Storage Mater..

[18] Lin M.C., Gong M., Lu B., Wu Y.P., Wang D.-Y., Guan M., Angell M., Chen C., Yang J., Hwang B. (2015). An ultrafast rechargeable aluminium-ion battery. Nature.

[19] Hong H., Liu J., Huang H., Etogo C.A., Yang X., Guan B., Zhang L. (2019). Ordered Macro-Microporous Metal-Organic Framework Single Crystals and Their Derivatives for Rechargeable Aluminum-Ion Batteries. J. Am. Chem. Soc..

[20] Huang C., Yang Y., Li M., Qi X., Pan C., Guo K., Bao L., Lu X. (2024). Ultrahigh Capacity from Complexation-Enabled Aluminum-Ion Batteries with C_70_ as the Cathode. Adv. Mater..

[21] Wang L., Zhu G., Lin Y., Wang Y., Zhu Q., Dai Z. (2023). MOF-derived hierarchical porous carbon octahedrons for aluminum-ion batteries. Carbon.

[22] Zhang Y., Zhang B., Li J., Liu J., Huo X., Kang F. (2021). SnSe nano-particles as advanced positive electrode materials for rechargeable aluminum-ion batteries. Chem. Eng. J..

[23] Ma D., Li J., Li H., Yuan D., Ji Z., Manawan M., de León Albarran C.P., Wu C., Pan J.H. (2024). Progress of Advanced Cathode Materials of Rechargeable Aluminum-Ion Batteries. Energy Mater. Adv..

[24] Zhang Y., Wen G., Fan S., Chu Y., Li S., Xu B., Zhang J. (2019). Phenolic hydroxyl functionalized partially reduced graphene oxides for symmetric supercapacitors with significantly enhanced electrochemical performance. J. Power Sources.

[25] Hu Z., Zhang H., Wang H., Zhang F., Li Q., Li H. (2020). Nonaqueous Aluminum Ion Batteries: Recent Progress and Prospects. ACS Mater. Lett..

[26] Jia B.E., Thang A.Q., Yan C., Liu C., Lv C., Zhu Q., Xu J., Chen J., Pan H., Yan Q. (2022). Rechargeable Aqueous Aluminum-Ion Battery: Progress and Outlook. Small.

[27] Canever N., Hughson F.R., Nann T. (2020). Solid-Electrolyte Interphases (SEI) in Nonaqueous Aluminum-Ion Batteries. ACS Appl. Energy Mater..

[28] Kaveevivitchai W., Huq A., Wang S., Park M.J., Manthiram A. (2017). Rechargeable Aluminum-Ion Batteries Based on an Open-Tunnel Framework. Small.

[29] Xue S., Li K., Lin Z., Zhang K., Zheng J., Zhang M., Shen Z. (2022). Rechargeable aluminum-ion battery based on interface energy storage in two-dimensional layered graphene/TiO_2_ electrode. Mater. Today Sustain..

[30] Wang Y., Zhang Z., Yuan F., Wang B. (2024). Design and modification of carbon-based materials for high energy density non-aqueous aluminum ion batteries: A review. J. Power Sources.

[31] Mohammad A., Köhler T., Biswas S., Stöcker H., Meyer D.C. (2023). A Flexible Solid-State Ionic Polymer Electrolyte for Application in Aluminum Batteries. ACS Appl. Energy Mater..

[32] Jin J., Zhang R., Zhi X., Liu D., Wang Y., Feng Z., Sun T. (2024). Aluminum vacancy-rich MOF-derived carbon nanosheets for high-capacity and long-life aqueous aluminum-ion battery. EcoEnergy.

[33] Smajic J., Alazmi A., Wehbe N., Costa P. (2021). Electrode-Electrolyte Interactions in an Aqueous Aluminum-Carbon Rechargeable Battery System. Nanomaterials.

[34] Al-Odayni A.B., Alsubaie F.S., Abdu N.A.Y., Al-Kahtani H.M., Saeed W.S. (2023). Adsorption Kinetics of Methyl Orange from Model Polluted Water onto N-Doped Activated Carbons Prepared from N-Containing Polymers. Polymers.

[35] Zhou Q., Zhang Z., Cai J., Liu B., Zhang Y.-L., Gong X., Sui X., Yu A., Zhao L., Wang Z.-B. (2020). Template-guided synthesis of Co nanoparticles embedded in hollow nitrogen doped carbon tubes as a highly efficient catalyst for rechargeable Zn-air batteries. Nano Energy.

[36] Kopecká J., Kopecký D., Vrňata M., Fitl P., Stejskal J., Trchová M., Bober P., Morávková Z., Prokeš J., Sapurina I. (2014). Polypyrrole nanotubes: Mechanism of formation. RSC Adv..

[37] Xu L., Li S., Mao H., Zhang A., Cai W., Wang T., Zhao Z. (2021). An advanced necklace-like metal organic framework with an ultrahighly continuous structure in the membrane for superior butanol/water separation. J. Mater. Chem. A.

[38] Yang W., Lu H., Cao Y., Jing P., Hu X., Yu H. (2020). A flexible free-standing cathode based on graphene-like MoSe_2_ nanosheets anchored on N-doped carbon nanofibers for rechargeable aluminum-ion batteries. Ionics.

[39] Ramli Z.A.C., Pasupuleti J., Kamarudin S.K., Zainoodin A.M., Isahak W., Koh S.P., Kiong S.T. (2024). Harnessing the Potential of Hollow Graphitic Carbon Nanocages for Enhanced Methanol Oxidation Using PtRu Nanoparticles. Polymers.

[40] Zhang J., Zhang L., Zhao Y., Meng J., Wen B., Muttaqi K.M., Islam M.R., Cai Q., Zhang S. (2022). High-Performance Rechargeable Aluminum-Ion Batteries Enabled by Composite FeF_3_@Expanded Graphite Cathode and Carbon Nanotube-Modified Separator. Adv. Energy Mater..

[41] Ke Q., Liu Y., Xiang R., Zhang Y., Du M., Li Z., Wei Y., Zhang K. (2022). Nitrogen-Doped Porous Core-Sheath Graphene Fiber-Shaped Supercapacitors. Polymers.

[42] Debnath S., Horscheck-Diaz M., Searles D.J., Hankel M. (2021). Carbon nitrides as cathode materials for aluminium ion batteries. Carbon.

[43] Chu W., Zhang X., Zhu F., Li S., Fu Y., Yu H. (2023). Organopolysulfides as high-performance cathode materials for rechargeable aluminum-ion batteries. J. Mater. Sci. Technol..

[44] Yuan D., Zhao J., Manalastas W., Kumar S., Srinivasan M. (2020). Emerging rechargeable aqueous aluminum ion battery: Status, challenges, and outlooks. Nano Mater. Sci..

[45] Wang Y., Cai J., Han T., Hu C., Zhu Y., Li J., Liu J. (2022). In-situ growing polyaniline nano-spine array on FeVO_4_ nanobelts as high-performance rechargeable aluminum-ion battery cathode. Appl. Surf. Sci..

[46] Huang W., Zhang K., Yuan B., Yang L., Zhu M. (2022). Predominant intercalation of H^+^ enables ultrahigh rate capability of oxygen deficient MoO_3_ for aqueous Al-ion batteries. Energy Storage Mater..

[47] Cang R., Song Y., Ye K., Zhu K., Yan J., Yin J., Wang G., Cao D. (2020). Preparation of organic poly material as anode in aqueous aluminum-ion battery. J. Electroanal. Chem..

[48] Xu J., Jiang P., Liang X., Tian R., Liu Y. (2021). Nitrogen-doped porous carbon as cathodes for aluminum-ion batteries. Fuller. Nanotub. Carbon Nanostruct..

[49] Thanwisai P., Chaiyapo N., Phuenhinlad P., Kanaphan Y., Nash J., Chotsuwan C., Klamchuen A., Wang Y., Nann T., Meethon N. (2022). Mesoporous and defective activated carbon cathode for AlCl_4_^-^ anion storage in non-aqueous aluminium-ion batteries. Carbon.

[50] Muñoz-Torrero D., Molina A., Palma J., Ventosa E., Marcilla R. (2020). Widely commercial carbonaceous materials as cathode for Al-ion batteries. Carbon.

[51] Guan W., Gu B., Tu J., Wang Z., Zhang P., Meng L., Jiao S. (2024). Stable Low-Temperature Al Batteries Enabled by Integrating Polydopamine-Derived N-Doped Carbon Nanospheres with Flake Graphite. Small.

[52] Tu J., Wang J., Li S., Song W.L., Wang M., Zhu H., Jiao S. (2019). High-efficiency transformation of amorphous carbon into graphite nanoflakes for stable aluminum-ion battery cathodes. Nanoscale.

[53] Ren M., Xu P., Zhou Y., Wang Y., Dong L., Zhou T., Chang J., He J., Wei X., Wu Y. (2022). Stepwise artificial yarn muscles with energy-free catch states driven by aluminum-ion insertion. ACS Nano.

