# Supplementary material for: Polypyrrole-Derived Nitrogen-Doped Tubular Carbon Materials as a Promising Cathode for Aqueous Aluminum-Ion Batteries
Source: Polymers (Basel). 2024 Nov 25;16(23):3276. doi: 10.3390/polym16233276 (PMC11644632; doi:10.3390/polym16233276)
Supplement: Supplementary file 1 [file polymers-16-03276-s001.zip › polymers-3324362-supplementary.pdf]

Supporting information

Polypyrrole-derived Nitrogen-doped Tubular Carbon Materials as a Promising Cathode for Aqueous Aluminum-ion Batteries

Xiaoming Zhou[a], Xiaolei Li[a], Jiaming Duan[a], Lihao Zhang[a], Xinyu Mo[a], Qing Wu[a], Yang Liu[a, *], Guohui Yuan[b, *], Miaosen Yang[a, *]

[a] School of chemical engineering, Northeast Electric Power University, Jilin 132012, P. R. China.

[b] School of chemistry and chemical engineering, Harbin Institute of Technology, Harbin 150001, P. R. China.

*Corresponding author. E-mail address: lyiuang@163.com (Yang Liu); yghhit@163.com (Guohui Yuan); ymiaosen@163.com

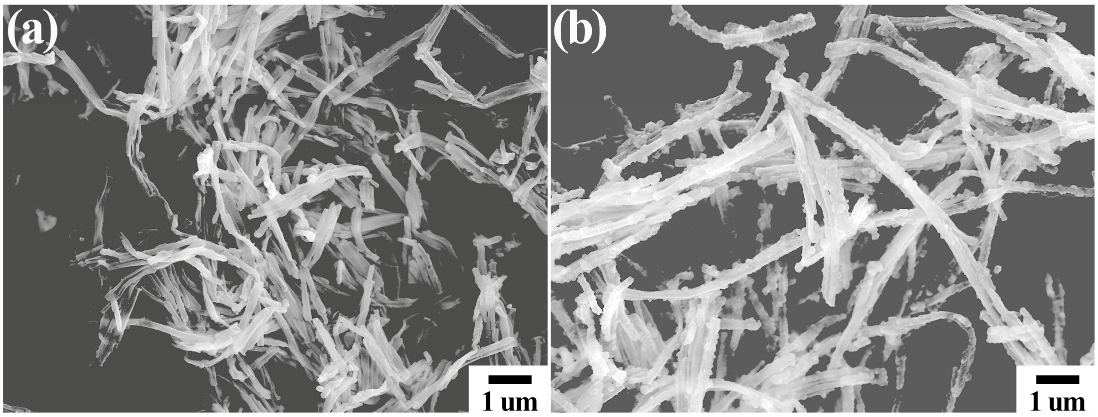

Figure S1. (a) SEM image for tubular polypyrrole polymer precursor; (b)SEM image of tubular carbon material.

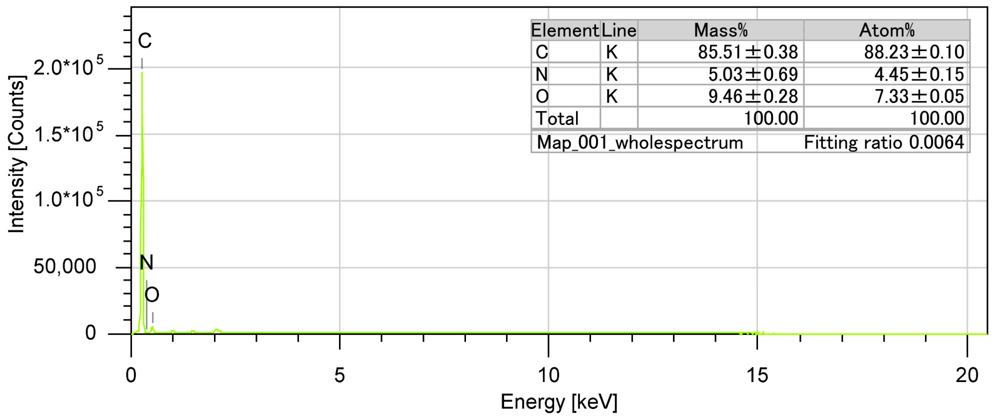

Figure S2. SEM-EDS elemental analysis of nitrogen-doped tubular carbon materials.

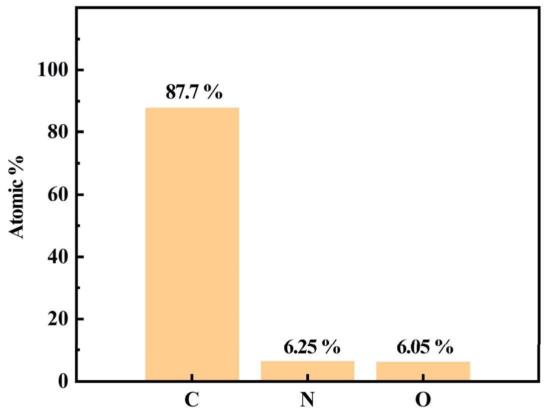

Figure S3. XPS elemental analysis of nitrogen-doped tubular carbon materials.

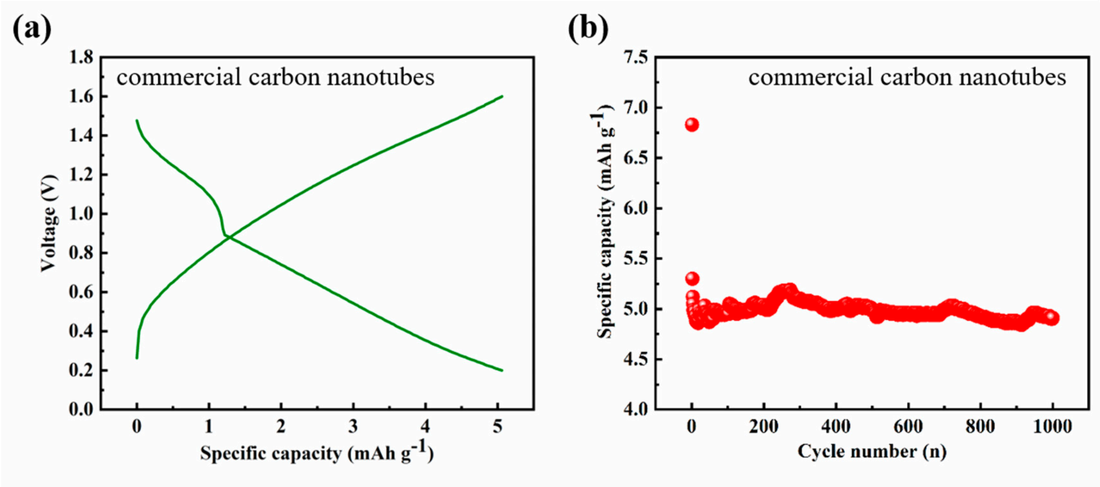

Figure S4. Electrochemical performance of untreated commercial carbon nanotubes as the cathode for aluminum-ion battery. (a) typical GCD curve, (b) cycling performance at 50 mA g^{-1}.
